# Acute myeloid leukemia and pregnancy: clinical experience from a single center and a review of the literature

**DOI:** 10.1186/s12885-017-3436-9

**Published:** 2017-06-23

**Authors:** Nicola Stefano Fracchiolla, Mariarita Sciumè, Francesco Dambrosi, Francesca Guidotti, Manuela Wally Ossola, Giovanna Chidini, Umberto Gianelli, Daniela Merlo, Agostino Cortelezzi

**Affiliations:** 1 Oncohematology Unit, Fondazione IRCCS Ca’ Granda Ospedale Maggiore Policlinico and University of Milan, Via Francesco Sforza, 35, 20122 Milan, Italy; 2 Gynecology and Obstetrics Unit, Fondazione IRCCS Ca’ Granda Ospedale Maggiore Policlinico, Via Francesco Sforza, 35, 20122 Milan, Italy; 3 Anesthesiology Unit, Fondazione IRCCS Ca’ Granda Ospedale Maggiore Policlinico and University of Milan, Via Francesco Sforza, 35, 20122 Milan, Italy; 4 Division of Pathology, Department of Pathophysiology and Transplantation, Fondazione IRCCS Ca’ Granda Ospedale Maggiore Policlinico and University of Milan, Via Francesco Sforza, 35, 20122 Milan, Italy

**Keywords:** Acute myeloid leukemia, Pregnancy, Chemotherapy

## Abstract

**Background:**

Acute myeloid leukemia (AML) accounts for more than two thirds of leukemia during pregnancy and has an incidence of 1 in 75,000 to 100,000. Its clinical management remains a challenging therapeutic task both for patient and medical team, given to the therapy-attributable risks for mother and fetus and the connected counseling regarding pregnancy continuation.

**Methods:**

We provided a review of updated literature and a comprehensive description of five maternal/fetal outcomes of AML cases diagnosed concomitantly to pregnancy and treated at our Institution from 2006 to 2012.

**Results:**

Median age at AML diagnosis was 32 years (31–39). One diagnosis was performed in first trimester and the patient asked for therapeutic abortion before starting chemotherapy. Three cases were diagnosed in second/third trimester; in one case leukemia was diagnosed concomitantly with intrauterine fetal death, while the remaining two patients continued pregnancy and delivered a healthy baby by cesarean section. In only one of these two cases chemotherapy was performed during pregnancy (at 24 + 5 weeks) and consisted of a combination of daunorubicine and cytarabine. Therapy was well tolerated and daily fetus monitoring was performed. After completion of 30 weeks of gestation a cesarean section was carried out; the newborn had an Apgar score of 5/1'-7/5'-9/10', oxygen therapy was temporarily given and peripheral counts displayed transient mild leukopenia. One patient had diagnosis of myelodysplastic syndrome rapidly progressed to AML after delivery. Four out of the 5 described women are currently alive and disease-free. Three children were born and long-term follow-up has shown normal growth and development.

**Conclusions:**

The treatment of AML occurring during pregnancy is challenging and therapeutic decisions should be taken individually for each patient. Consideration must be given both to the immediate health of mother and fetus and to long-term infant health. Our series confirmed the literature data: fetal toxicity of cytostatic therapy clusters during the first trimester; while chemotherapy can be administered safely during second/third trimester and combination of daunorubicin and cytarabine is recommended for induction.

## Background

Cancer is diagnosed in about 0.07% to 0.1% of pregnancies and represents the second most common cause of maternal death after gestation-related vascular complications [1–7].

Pregnant women showed the same cancer frequency and localization when compared to nonpregnant ones of the same age [2]. The majority of described cases are solid tumors; oncohematologic diseases represent 25% of all cancers diagnosed during pregnancy [1–7]. The incidence of leukemia during gestation is estimated to range from 1 in 75,000 to 100,000 pregnancies, although its real epidemiology remains poorly defined [1–7]. Acute myeloid leukemia (AML) accounts for two-thirds of all cases, while acute lymphoid leukemia, chronic leukemia or myelodysplastic syndrome are rarely described [2–6]. Acute leukemia is usually reported during the second and third trimesters of pregnancy, accounting for 37% and 40% of the cases respectively [3–8]. In the first trimester the incidence is generally estimated around 23%, even if an underreporting bias caused by spontaneous pregnancy termination could not be excluded [3–8].

The rarity of hematologic neoplasia during pregnancy has precluded prospective controlled trials; hence, data are restricted to retrospective series and case reports, with the related difficulties in establishing management guidelines. Evidence obtained so far are insufficient for determining a causative association between pregnancy and hematological cancers [4].

Appropriate leukemia treatment has significantly improved survival and the central role of specific therapy during gestation has been emphasized while clinical experience accumulates [1, 7–16].

The therapeutic approaches to leukemia during pregnancy may be conditioned by several variables, including the gestational age at diagnosis, the clinical and biological disease characteristics, and the potential drug toxicity on mother and child [16–24].

Herein, we describe fetal and maternal outcomes of five cases of acute myeloid leukemia occurring during pregnancy consecutively diagnosed and treated at our Institution in order to make useful suggestions in the management of acute myeloid leukemia during pregnancy.

## Methods

Five patients with AML, diagnosed during or immediately after pregnancy, were identified in the institutional database between 2006 and 2012.

AML diagnosis was performed according to World Health Organization (WHO) 2008 diagnostic criteria for the cases diagnosed from 2008 to 2012 [25], and according to WHO 2001 for those diagnosed before 2008 [26].

Baseline clinical data, pregnancy characteristics, treatment modalities, efficacy, safety and disease courses were extracted by carefully reviewing all clinical data-sources represented by original paper-based patient and electronic records (medical and nursing paper-notes, blood chemistry, blood counts and coagulation tests, histological reports, echocardiography, radiological examinations and other diagnostic procedures).

Histological analysis of placenta was performed by local pathologists for each patient.

All procedures followed were in accordance with the ethical standards of the Helsinki Declaration of 1975, as revised in 2008.

## Results

A summary of the therapeutic regimen, gestational age, delivery method, mothers’ and infants’ outcomes is presented in Table 1.

**Table 1:** Characteristics of cases of AML treated during pregnancy at our Istitution

Clinical case 1	
Mother’s age, years	31
Gestational age, weeks	14
Induction chemotherapy	-Induction: daunorubicin 50 mg/m^2^ (days 1, 3, 5) and cytosine arabinoside 3 g/m^2^ (days 1–5) -Reinduction: fludarabine 30 mg/m^2^ (days 1–4), cytosine arabinoside 2 g/m^2^ (days 1–4) and idarubicin 12 mg/m^2^ (days 1–4)
Consolidation treatment	-Cytosine arabinoside 3 g/m^2^ twice a day (days 1–3-5) -Myeloablative sibling HSCT (conditioning regimen: busulfan 0,8 mg/kg three times a day days −9 -8 -7 -6 and cyclophosphamide 60 mg/kg days −3 -2)
Maternal outcome	CR
Fetal outcome	Surgical abortion at AML diagnosis
Delivery	-
Clinical case 2	
Mother’s age (years)	36
Gestational age (weeks)	32
Induction chemotherapy	Daunorubicin 50 mg/m^2^ (days 1, 3, 5) and cytosine arabinoside 3 g/m^2^ (days 1–5)
Consolidation treatment	-Cytosine arabinoside 3 g/m^2^ twice a day (days 1–4), daunorubicin 50 mg/m^2^ (days 5–6) -Cytosine arabinoside 300 mg/m^2^ (days 1–5), daunorubicin 50 mg/m^2^ (days 1–2) -Cytosine arabinoside 300 mg/m^2^ (days 1–5), etoposide 150 mg/m^2^ (days 1–3) -Cytosine arabinoside 1 g/m^2^ twice a day (days 1, 3, 5) -Autologous HSCT (conditioning regimen: cytosine arabinoside 3 g/m^2^ twice a day, cyclophosphamide 60 mg/kg twice a day for two days, 1000 cGy of total body irradiation)
Maternal outcome Fetal outcome Delivery	CR Live birth, normal child Cesarean at AML diagnosis
Clinical case 3	
Mother’s age (years)	32
Gestational age (weeks)	26
Induction chemotherapy	Cytosine arabinoside 2 g/m^2^ twice a day (days 1, 2, 8, 9) and idarubicin 18 mg/m^2^ (days 2, 3, 10) 6 weeks after delivery when MDS progressed to AML
Consolidation treatment	-Cytosine arabinoside 100 mg/m^2^ (days 1–7), idarubicin 10 mg/m^2^ (days 1–3) -Myeloablative sibling HSCT (conditioning regimen: busulfan 0.8 mg/kg three times a day, days −9 -8 -7 -6, cyclophosphamide 60 mg/kg, days −3 -2)
Maternal outcome Fetal outcome Delivery	CR Live birth, normal child Cesarean at 32 + 2 weeks after administration of betamethasone
Clinical case 4	
Mother’s age (years)	34
Gestational age (weeks)	31
Induction chemotherapy	Cytosine arabinoside 3 g/m^2^ (days 1–5) and daunorubicin 50 mg/m^2^ (days 2, 4, 5)
Consolidation treatment	-Cytosine arabinoside 3 g/m^2^ twice a day (days 1–4), daunorubicin 50 mg/m^2^ (days 5–6) −2 cycles of cytosine arabinoside 3 g/m^2^ twice a day (days 1–3-5)
Maternal outcome Fetal outcome Delivery	CR Intrauterine fetal death Cesarean at AML diagnosis
Clinical case 5	
Mother’s age (years)	39
Gestational age (weeks)	24 + 5
Induction chemotherapy	Cytosine arabinoside 100 mg/m^2^ (days 1–7) and daunorubicin 40 mg/m^2^ (days 1–3)
Consolidation treatment	4 cycles of cytosine arabinoside 3 g/m^2^ twice a day (days 1–3-5)
Maternal outcome	CR; death for septic shock during the fourth and last consolidation cycle with high dose cytosine arabinoside
Fetal outcome Delivery	Live birth, normal child Cesarean at 30 weeks after administration of betamethasone

*CR* complete remission, *HSCT* hematopoietic stem cell transplantation

### Clinical case 1

A 31 years old woman from Sri Lanka at 14 weeks gestational age, during a routine control, showed a decrease of body weight and leucopenia [white blood cell (WBC) 3.65 × 10^9^/L]. The mother was pregnant for the second time, with a caesarian section performed 3 years before for a cervical dystocia. The bone marrow aspirate demonstrated a diagnosis of AML without maturation (WHO 2008). Cytogenetic analysis showed a normal karyotype. Fluorescence in situ hybridization (FISH) karyotyping was negative for inv.(16) and t(8;21). The patient displayed D835 FLT3 mutation in the tyrosin-kinase domain.

Five days after multidisciplinary consulting the patient asked for surgical abortion. The procedure was performed without complications and after one week the patient started chemotherapy.

Induction chemotherapy with daunorubicin and cytosine arabinoside was performed, and a partial remission was obtained (Table 1). A second induction with FLAG-IDA regimen was administered, obtaining complete remission (CR). Consolidation therapy was completed with allogeneic myeloablative hematopoietic stem cell transplantation (HSCT) from an HLA identical sister (Table 1). The patient is alive and well with a follow up of 54 months.

### Clinical case 2

A 36 years old Caucasian woman at 32 weeks gestational age at her fourth pregnancy, was referred to our Center for a suspected HELLP syndrome. On admission peripheral blood counts and coagulation tests displayed WBC 3.4 × 10^9^/L, hemoglobin (Hb) 8.2 g/dl, platelets 27 × 10^9^/L, fibrinogen 156 mg/dl, D-Dimer 13,675 μg/mL. Lactate dehydrogenase was increased (689 U/L). An urgent cesarean section was performed and a morphologic normal male weighting 2290 g was born. The bone marrow aspirate demonstrated a diagnosis of acute monoblastic leukemia (WHO 2001). Cytogenetic analysis was unsuccessful for absence of metaphases. FISH was negative for inv.(16) and t(8;21). Induction chemotherapy with daunorubicin and cytosine arabinoside was performed and it was complicated by disseminated intravascular coagulation treated with fresh frozen plasma infusions. CR was documented at bone marrow examination. Consolidation treatment is reported in detail in Table 1. The patient is alive and in good health after a follow up of 96 months.

### Clinical case 3

A 32 years old Caucasian woman showed rapid decrease of peripheral blood counts over a three months period. The patient underwent a previous cesarean section for breech presentation and a previous spontaneous abortion at 9 gestational weeks. On admission she was at the 26th gestational week and showed severe pancytopenia (Hb 8.6 g/dl, platelets 81 × 10^9^/L, WBC 3.32 × 10^9^/L). At ultrasound examination fetus had regular morphology and normal growth. We decided for a watch and wait strategy, with close monitoring of fetal growth by ultrasound examination and cardiotocography, and performing filtered hemocomponents transfusions to maintain Hb >8 g/dl and platelets >20 × 10^9^/L. At the same time, betamethasone 12 mg i.m. was administered for fetal lungs maturation.

The bone marrow aspirate demonstrated a diagnosis of refractory anemia with excess of blasts-1 (WHO 2008), with a intermediate-1 IPSS risk class (score 1) [27]. 

The pregnancy was terminated by elective cesarean section at 32 + 2 weeks of gestation without complications. Placenta histological examination displayed normal findings. The first evaluation of bone marrow and flow-cytometry analysis 6 weeks after delivery showed disease progression to AML with myelodysplasia-related changes (WHO 2008). Cytogenetic analysis showed a normal karyotype. Induction chemotherapy with daunorubicin and cytosine arabinoside was performed obtaining a CR at bone marrow examination. Consolidation treatment is reported in detail in Table 1. Allogeneic myeloablative HSCT was performed from an HLA identical brother. The patient is alive and in good health after a follow up of 36 months after transplantation without major complications.

### Clinical case 4

A 34 years old Caucasian woman at 31 weeks gestational age, developed important abdominal pain, asthenia and fever. The patient had in her obstetric history a previous abortion, an unsuccessful medically assisted fertilization procedure, and a spontaneous pregnancy with elective cesarean delivery. On admission, ultrasonography showed an in utero still birth. An urgent cesarean section was performed and a death fetus weighing 1865 g was extracted. No surgical adverse event was reported.

Peripheral blood counts and coagulation tests displayed WBC 5.32 × 10^9^/L, platelets 49 × 10^9^/L, D-Dimer 4689 μg/ml, fibrinogen 167 mg/dl. Lactate dehydrogenase was increased (1008 U/L).

**Fig. 1:**
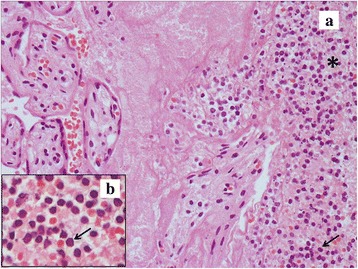
**a** Clinical case 4, acute myeloid leukemia cells limited to intervillous maternal space, which is indicated by the asterisk. Hematoxylin-eosin staining. The area pointed out by the arrow is magnified in Fig. 1(B). **b** Myeloblasts show mild size variation, have pale to slightly basophilic agranular cytoplasm and uniform large nuclei with condensed chromatin. Blast cells are pointed out by the arrow

**Fig. 2:**
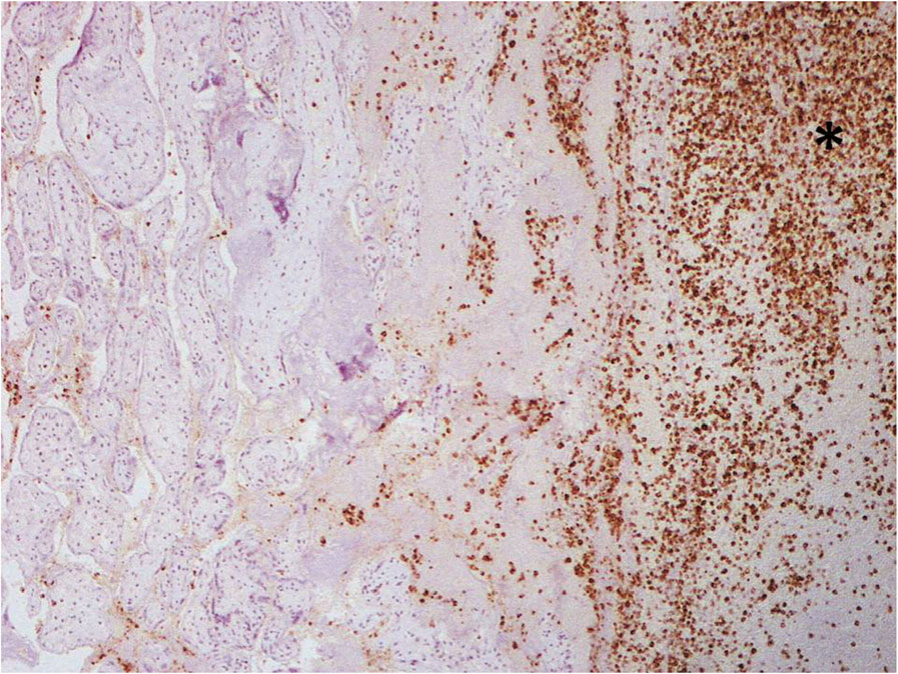
Clinical case 4, immunohistochemical staining for myeloperoxidase highlights myeloblasts in the intervillous maternal space, which is pointed out by the asterisk

Histological analysis of placenta revealed multiple recent infarcts, intraplacentar ematomas with infarctions of surrounding parenchima and not recent subchorial thromboses, with regular villous maturation and branching of the cotyledons appropriate for 31 gestational weeks. Acute myeloid leukemia cells (MPO + CD34-CD7-) were limited to intervillous maternal space (Figs. 1, 2). Bone marrow aspirate revealed a diagnosis of AML without maturation (WHO 2001). Cytogenetic was normal and FISH karyotyping was negative for t(15;17), inv.(16) and t(8;21). FLT3-ITD and NPM1 mutation were both present. Induction chemotherapy with daunorubicin and cytosine arabinoside was performed. The clinical course was complicated by fungal liver infection. After hepatitis resolution, CR was documented at bone marrow examination. Consolidation treatment is reported in detail in Table 1. The patient is alive and in good health after a follow up of 53 months.

### Clinical case 5

A 39 years old Caucasian woman was referred to our Institution for severe pancytopenia at routine peripheral blood examination. Blood counts showed WBC 16 × 10^9^/L, Hb 5.1 g/dl, platelets 47 × 10^9^/L. Myeloid blasts in peripheral blood were 74%. Coagulation parameters were normal. She was at her second pregnancy with a previous low transverse caesarian section for macrosomia after intracytoplasmatic sperm injection. At the moment of the referral, she was at 24 + 5 weeks of spontaneous conception pregnancy by menstrual age. Ultrasound examination described a single fetus at 22 + 4 weeks gestation with normal anatomy, amniotic fluid and umbilical artery doppler systolic–diastolic ratio. Intramuscular betamethasone was administered for fetal lung maturation. Bone marrow aspirate revealed a diagnosis of acute monoblastic leukemia (WHO 2008). FISH analysis was positive for inv.(16). The patient was transferred to the intensive care unit for strict monitoring.

Induction chemotherapy with daunorubicin and cytosine arabinoside was performed. Daily ultrasound and tocographic monitoring of fetal status was performed. CR was documented at bone marrow examination. After the completion of 30 weeks of gestation, a cesarean section under general anesthesia was performed.

A morphologic normal male weighting 1495 g was born. Apgar score at birth was 5/1′- 7/5′ - 9/10′. Oxygen therapy was given to the newborn and he was transferred to the neonatal intensive care unit.

Four days after cesarean section the patient was transferred to the oncohematology ward to continue leukemia treatment. During the last consolidation cycle, the woman died for a septic shock causing multiorgan failure.

### Children outcomes

In the described population, one case of AML was diagnosed concomitantly with intrauterine fetal death; only one patient asked for a therapeutic abortion before starting chemotherapy. The three remaining patients continued a regular pregnancy and delivered a healthy baby.

Only one child was exposed to chemotherapy during the gestational period (clinical case 5). The newborn needed oxygen therapy immediately after birth and suffered of a mild leukopenia, which resolved spontaneously after a few days. He is 5 years old and currently followed at our Institution according to the local clinical guidelines. All tests performed testified a normal neurodevelopment and no impairments to general health and growth, including a normal cardiac outcome by echocardiography. The other two children were born by a cesarean delivery at AML diagnosis and before the start of a chemotherapy program; physical evaluations and standard blood examinations were normal at birth and at the time of last available follow-up when they were 1 and 5 years old, respectively.

## Discussion

We report here the diagnosis, treatment and outcome of five consecutive cases of acute myeloid leukemia during pregnancy, occurred from 2006 to 2012 in a single Hematologic Unit.

The clinical picture of AML presenting during pregnancy is similar to that of non-pregnant women, and the diagnostic criteria are those defined in the WHO classification of myeloid neoplasms [25, 26]. The overlap of some common symptoms reported during pregnancy, such as fatigue and shortness of breath, or physiologic alteration of peripheral blood counts, as anemia and thrombocytopenia, might delay the diagnostic suspicion and appropriate therapy. In this respect, the main differential diagnoses to consider are thrombotic microangiopathy, HELLP syndrome and cytopenias of deficiency or immune origin [7].

Acute leukemia may present with hyperleukocytosis, thromboses or disseminated intravascular coagulation, in the context of a gestational associated thrombogenic milieu [1–5]. Thrombosis could affect placental vessels, impairing fetal growth and survival [28, 29]. Intrauterine death may also be the result of hypoxia caused by placental ischaemic infarction and leukemic infiltration [1–5], as occurred in one of the patients described in this series.

Essential investigations for a correct differential diagnosis should include full blood count and blood film examination, vitamin B12, folate and ferritin measurement, coagulation and hemolysis screening, renal and liver function tests [7]. When a diagnosis of leukemia is suspected, marrow samples should be obtained and morphological, immunophenotypic, cytogenetic and molecular analyses performed to allow accurate sub-typing and correct prognostication, according to published guidelines [30].

AML associated to pregnancy is rare and only small patients series have been reported [1–15]. The anti-leukemic therapies during pregnancy may differ from golden standards, being conditioned by several variables, including the gestational age at diagnosis, the clinical and biological characteristic of the disease, and the potential drugs toxic effects on mother and child [6–15].

Fetal toxicity of cytostatic therapy clusters during the first trimester, while it declined dramatically during the second and third ones [1–7, 15–24]. During the first trimester chemotherapy results in fetal death in about 40% of cases, while the percentage is about 10% during the second trimester and almost the totality of infants who were exposed to chemotherapy in the third trimester were born alive with no major malformations, despite cases of growth retardation, intellectual impairment, reduced fertility and hematopoietic suppression have been reported [1–7, 15–24]. In most of the described cases, patients diagnosed during the first trimester underwent abortion and subsequently were treated with induction chemotherapy [6–15].

In the few cases treated with chemotherapy during the first trimester, poor pregnancy outcomes were reported with congenital anomalies and spontaneous abortions, while it was suggested that a prolonged delay in therapy initiation may be associated with a worse maternal outcome [6–15].

When a diagnosis of AML associated to pregnancy is made, a multidisciplinary team must be created to coordinate all the diagnostic and therapeutic steps, including the planning of a cesarean delivery before or between courses of chemotherapy and after maternal bone-marrow reconstitution, to avoid maternal and neonatal pancytopenia.

To minimize risks for mother and fetus, a near term cesarean delivery (>35–37 weeks) remains the main goal. Survival following delivery at or beyond 28 weeks gestation are >90% in most large centers, but even higher (>95%) if the delivery was at or beyond 32 weeks gestation [7].

If it is possible to postpone the initiation of the therapeutic program, a cesarean section should be planned before chemotherapy, or at peripheral blood counts recovery after the treatment cycle. Chemotherapy should not be administered after the 35th gestational week [1–7]. Intensive ultrasound monitoring is essential to document fetal growth, development, cardiac function and placental status, as well as tocographic daily assessment. When delivery is necessary at an earlier gestational age (before 34 weeks), a pharmacologic enhancement of fetal lung maturation by corticosteroids should be considered [7]. Breast feeding is contraindicated during the maternal anti-leukemic program [7].

The use of hydroxycarbamide before starting induction chemotherapy should be avoided except in cases of hyperleukocytosis (WBC greater then 100 × 10^9^/L) [7]. An alternative is leukapheresis, that allows rapid reduction of WBC counts in symptomatic patients. Large experiences with this procedure during AML in pregnancy are not available, but the few described cases demonstrated that it is a feasible emergency measure bridging to cytotoxic therapy [29]. The leukapheresis must be performed by expert personnel, and a careful monitoring of patient’s cardiocirculatory parameters, blood counts, coagulation status and electrolyte balance, together with fetus’ cardiac function (cardiotocography) must be guaranteed throughout the procedure. Continuous-flow cell separators should be preferably used to ensure the lowest extracorporeal volume, and saline or 5% human albumin is commonly used as replacement fluid, while fresh frozen plasma should be preferentially used in case of hemorrhagic features for coagulation factors deficiencies or intravascular coagulation [31].

A single chemotherapy cycle with daunorubicin and cytarabine is recommended for induction (daunorubicin 60 mg/m^2^ on days 1–3 and cytarabine 100 mg/m^2^ continuous infusion on days 1–7) [7]. The use of cytarabine at the same dosage, but for 10 days of infusion could be considered and it represents the standard regimen in the UK. [7] There are also no data to support the use of higher dose of daunorubicin and this approach is not recommended for increased toxicity [7]. Idarubicin, for its lipophilic characteristics, has an increased placental transfer and higher potential foetal toxicity, and therefore daunorubicin remains the anthracycline of choice [12, 16–20, 23]. It is highly advisable to monitor fetal cardiac function for anthracycline rare cardiotoxicity and limb morphogenesis for teratogenic potential of cytarabine (10%) [7, 16–24].

Standard consolidation therapy with the use of intermediate or high dose cytarabine is preferred [7, 30].

Acute promyelocytic leukemia (APL) represents a special situation. The information available on maternal and fetal outcomes in pregnant women with APL is limited. It could be a significant problem because it is usually associated with coagulopathy; platelet transfusions should be considered to maintain a platelet count of more than 30 × 10^9^/L and fresh frozen plasma for a fibrinogen level higher than 150 mg/dl. The management during the first trimester of pregnancy should be separated from that arising in the second/third semester. All-trans retinoic acid (ATRA) is the cornerstone of APL treatment, but it can be potential teratogenic and should be avoided during the first trimester. Similarly, arsenic trioxide has a high potential embryotoxicity and cannot be recommend at any stage of pregnancy. If the patient wish to preserve fetus, anthracycline alone is recommended during the first trimester. Treatment by ATRA alone during the second and the third trimester until complete remission achievement can be proposed, delaying the administration of chemotherapy after birth [32, 33].

Regarding the allowed supportive therapies during pregnancy, the first line antiemetic agent are the antihistamines, cyclizine and promethazine; metoclopromide may induce maternal dystonic reactions, therefore it should be considered as a second line drug. Ondasentron could be administered safely during the first trimester, while tropisetron is not permitted because malformations in animals were reported [7]. The use of granulocyte colony stimulating factor during gestation was rarely reported, but the available data seem to suggest its safe use in the second and third trimesters [34].

Penicillins were not associated to injurious fetal effects, with the exception of the combination amoxicillin and clavulonate. Aminoglycosides, quinolones, sulphonamides and tetracyclines are not permitted; while the main systemic antifungal drug safely used was amphotericin B or lipid derivates. If a transfusion is required, cytomegalovirus-negative blood products represent the first choice [7].

Based on the available data, pregnant women with AML treated with appropriate regimens by expert and multidisciplinary teams, might obtain outcomes similar to those of non pregnant woman, and the prognosis is worse only when appropriate treatment is significantly delayed [1–15].

In our series, induction treatment consisted of a combination of anthracyclines and cytarabine; in particular, the only patient treated before delivery received standard dosage of cytarabine and daunorubicin at a reduced dosage of 40 mg/m^2^ (days 1–3). With a median follow-up of 6 years from diagnosis, overall survival is of 80% and leukemia free survival is of 100%.

These results reproduce those observed for adolescents and young adults (AYA) AML. The reasons of the improved outcomes among AYA-AML, to which AML during pregnancy may be associated, in comparison to their older counterparts, include a multifactorial explanation, encompassing both patient/host (better performance status, few comorbidities, optimal intensive chemotherapy tolerance) and AML disease biology (reduced incidence of high-risk chromosomal abnormalities or multi-drug resistance expression) [35].

## Conclusions

The cases described here have represented for our institution a clinical challenge that forced us to build a multidisciplinary team involving hematologists, obstetricians, anesthesiologist and pediatricians/neonatologists, in order to properly manage all the phases of leukemia diagnosis and treatment, as well as those concerning pregnancy and newborn.

The available therapeutic options were proposed to, and the connected risks and alternatives were presented and discussed with, each patient, by the different specialists involved in the team, always collegially.

In those cases diagnosed in advanced gestational age, we postponed the leukemia treatment after delivery, performed in all cases by carefully programmed caesarean section.

In the only case occurring in the first trimester, the patient asked for therapeutic abortion. If chemotherapy is administered during pregnancy, extensive and continuous monitoring of fetus vital signs, cardiac function and congenital malformation is mandatory in order to promptly diagnose and treat emerging complications related to the disease or therapy. Cord blood stem cells should be collected and stored in the event that an irreversible bone marrow failure syndrome should develop in the fetus.

## Abbreviations

AML: acute myeloid leukemia
APL: acute promyelocytic leukemia
ATRA: all-trans retinoic acid
CR: complete remission
FISH: fluorescence in situ hybridization
FLT3-ITD: FLT3 internal tandem duplication
Hb: hemoglobin
HSCT: hematopoietic stem cell transplantation
WBC: white blood cell
WHO: World Health Organization.
